# Phylogeographic structure of the dwarf snakehead (*Channa gachua*) around Gulf of Tonkin: Historical biogeography and pronounced effects of sea‐level changes

**DOI:** 10.1002/ece3.8003

**Published:** 2021-08-17

**Authors:** Junjie Wang, Chao Li, Jiaqi Chen, Jujing Wang, Jinjin Jin, Shuying Jiang, Luobin Yan, Hung‐Du Lin, Jun Zhao

**Affiliations:** ^1^ Guangdong Provincial Key Laboratory for Healthy and Safe Aquaculture Guangdong Provincial Engineering Technology Research Center for Environmentally‐friendly Aquaculture Guangzhou Key Laboratory of Subtropical Biodiversity and Biomonitoring School of Life Science South China Normal University Guangzhou China; ^2^ The Affiliated School of National Tainan First Senior High School Tainan Taiwan

**Keywords:** *Channa gachua*, cryptic species, Hainan Island, phylogeography, S‐DIVA

## Abstract

Geological events, landscape features, and climate fluctuations have shaped the distribution of genetic diversity and evolutionary history in freshwater fish, but little attention has been paid to that around the Gulf of Tonkin; therefore, we investigated the phylogeographic structure of the dwarf snakehead (*Channa gachua*) on Hainan Island and mainland China, as well as two populations in Vietnam. We attempted to elucidate the origins of freshwater fish in South Hainan by incorporating genetic data from DNA markers on both the mitochondrial cytochrome *b* gene (cyt *b*) and the nuclear recombination‐activating gene 1 (RAG‐1). Mitochondrial phylogenetic analysis identified two major lineages (lineages A and B), which may represent separate species. Divergence data suggested that *C. gachua* populations diverged between 0.516 and 2.376 myr. The divergence of the two cryptic species is congruent with sea‐level rise, which subsequently isolated Hainan from the mainland. During the Pleistocene glaciations, the entire region of the Gulf of Tonkin and the Qiongzhou Strait became part of the coastal plain of the Asian continent, which might have resulted in the current distribution patterns and dispersal routes of *C. gachua* populations. The formation of three sublineages in lineage A indicated that the Gulf of Tonkin was a geographical barrier between Hainan Island and mainland China but not between Vietnam and Hainan Island. The results of this study may help to elucidate the origins of freshwater fish in South Hainan and the phylogeographic structure of *C. gachua*.

## Graphical abstract

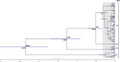

## 1 INTRODUCTION

Continental islands provide excellent opportunities for examining phylogeographic patterns in freshwater fish, with their biogeographic relationships that reflect historical rather than present‐day drainage connections (Han et al., 2019). The dispersal of primary freshwater fish will be strongly affected by geological events. Islands are typically separated from continents by the sea, and fluctuating sea levels may cause continental islands to connect and separate from the mainland repeatedly and provided a suitable route for species to migrate. Combined with climatic oscillations, local geological events play a role in driving genetic patterns leading to diversification, speciation, and biogeography. The South China Sea has fluctuated greatly and experienced considerable climatic changes during the Pleistocene when glacial cycles acted as a major driver shaping the present‐day diversity and distribution patterns of species.

Hainan Island is located off the south coast of China and is separated from mainland China to the north by the Qiongzhou Strait and from northern Vietnam to the west by the Gulf of Tonkin (Zhu, 2016). The topography of Hainan Island is diverse, with the Wuzhishan and Yinggeling Mountain Range (WY Range) approaching elevations of 1,800 m and serving as the core of the uplift. The topology of the island rises steeply from the central and southern regions and extends north to a wide plain. The four largest rivers on the island, the Nandu, Changhua, Wanquan, and Linshui Rivers, originate from the central mountainous area. A comparison of the freshwater fish species of Hainan Island, northern Vietnam, and mainland China around the Gulf of Tonkin (Beibu Gulf) showed that they were quite similar to each other. The withdrawal of the South China Sea during several inter‐ice ages, Gulf of Tonkin was exposed due to the decrease in sea level, might have led to the colonization and divergence of some fish populations in the coastal rivers of northern Vietnam and mainland China (Voris, 2000; Zhao et al., 2007; Zhou et al., 2017). According to previous phylogeographic studies, freshwater fishes as migrants probably moved between mainland China and island populations during low sea level via water system confluence (Chen et al., 2007; Yang et al., 2016). Similar phenomena were also found in the phylogeographic studies of many freshwater fishes in the drainages of Hainan Island or on both sides of the Qiongzhou Strait (e.g., *Glyptothorax hainanensis*, see Chen et al., 2007; *Garra orientalis*, see Yang et al., 2016; genus *Opsariichthys,* see Lin et al., 2016; and *Opsariichthys hainanesi*, see Zhang et al., 2020).

In addition, some studies have shown that freshwater fish on Hainan Island may have originated in northern Vietnam in the west, rather than in southern China in the north (Lin et al., 2010). Our previous studies have shown that some freshwater fish in southwestern Hainan Island are more closely related to freshwater fish from the Red River in mainland China (Zhang et al., 2020). Moreover, Lin et al. (2010) examined the population structure of Reeves's butterfly lizard (*Leiolepis reevesii*) and proposed the migration route of *L. reevesii* from Vietnam to Hainan followed by a second wave of dispersal from Hainan to mainland China. Accordingly, the species of Hainan Island might originate from the north (China) or west (Vietnam). However, to the best of our knowledge, there is no research on the population relationship between North Vietnam and Hainan Island. Therefore, we propose investigating whether the freshwater fish in southwestern Hainan Island and the freshwater fishes of the Red River in mainland China belong to the same fish zoogeographical region. The above studies reflect that the freshwater fishes of Hainan Island may not have a single origin, but due to the lack of information on freshwater fishes in northern Vietnam, the relationship between freshwater fish in Vietnam and Hainan has not been studied. Therefore, the phylogenetic process of freshwater fish on Hainan Island has not been effectively and reasonably explained. To date, there have been no comparable data on freshwater fish from geographically separated populations between Hainan Island and the Red River, although such data will provide additional insights into the phylogeographic history of the species and contemporary factors shaping the population genetic structure and supporting the implementation of effective management and conservation strategies for freshwater fish.

*Channa gachua* (Hamilton, 1927) belongs to the order Anabantiformes, family Anabantiformes, and genus *Channa*, and is a freshwater fish restrictively distributed in various water systems of the Hainan Island zoogeographical region and Nujiang–Lancangjiang zoogeographical region in China but is primarily distributed in Nepal, the Indian subcontinent, Afghanistan, and Southeast Asia abroad (Chu & Chen, 1990). This species is usually found in hill streams, while adults inhabit medium to large rivers, brooks, rapid‐running mountain streams, and stagnant water bodies, including sluggish flowing canals (Kottelat et al., 1998; Taki et al., 1978). The species is not distributed in the coastal waters of southern China. According to this distribution pattern, *C. gachua* is an ideal fish for inferring the biological consequences of historical biogeography in this area. In the present study, to address the abovementioned problems, we used the mitochondrial DNA (mtDNA) cytochrome *b* gene (cyt *b*) and nuclear gene rag 1 region (RAG‐1) to establish the phylogeographic patterns in South China and northern Vietnam. There are two major questions investigated by our study: (1) What is the genetic diversity and genetic structure of *C. gachua*? and (2) How did *C. gachua* colonize the rivers of different geographical districts on Hainan Island? Whether the freshwater fish in southwestern Hainan Island and the freshwater fishes of the Red River in mainland China belong to the same fish zoogeographical region has not been elucidated. The results of this study may help to elucidate the population genetic structure of *C. gachua* that may contribute to conservation, management, and sustainable utilization of freshwater fish on Hainan Island and may also help to provide context for other threatened endemic fish species in the same or similar river systems.

## 2 MATERIALS AND METHODS

### 2.1 Sampling and molecular methods

**FIGURE 1:**
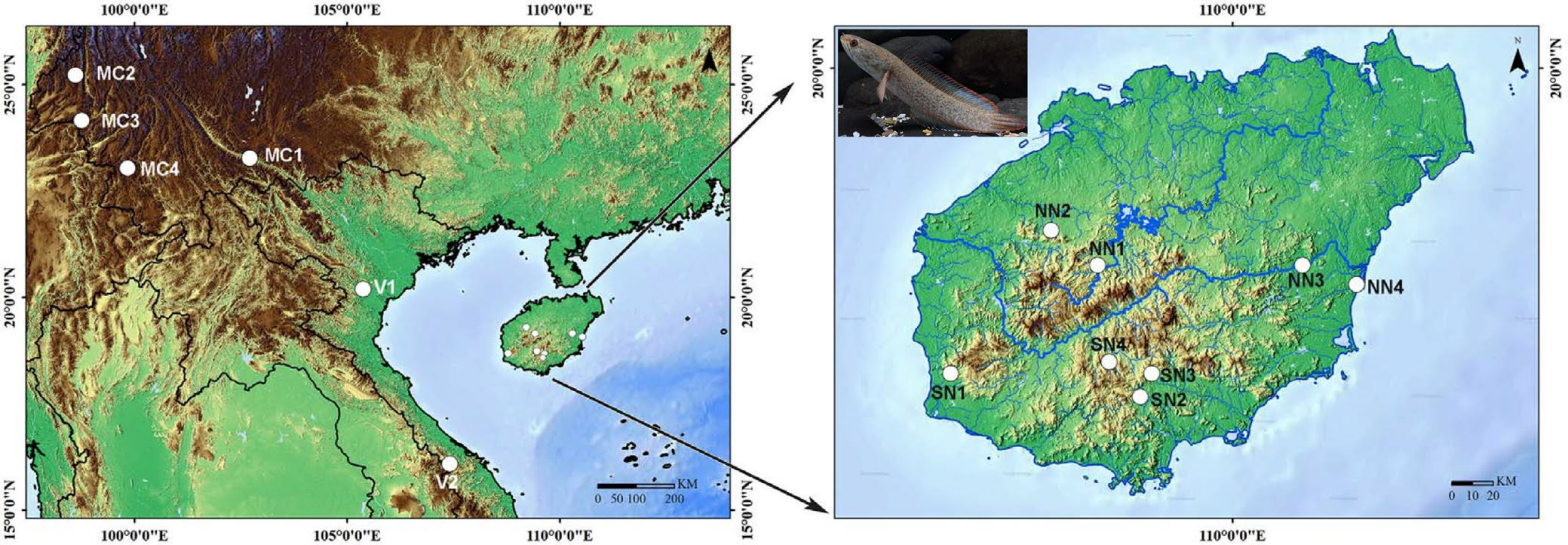
Map showing the 14 sampling localities of *Channa gachua* used in this study. Collection sites (circles) correspond to locations given in the text and Table 1

A total of 336 individuals were collected from twelve localities on Hainan Island and mainland China and two populations in Vietnam between July 2017 and December 2019 (Table 1; Figure 1). Samples were collected from the field sites with seines, fatally anesthetized with MS‐222 (Sigma, St. Louis, MO), and fixed and stored in 95% ethanol. Sampling information and localities are provided in Table 1 and Figure 1. All animal experiments were lodged in the laboratory of Jun Zhao, Guangzhou Key Laboratory of Subtropical Biodiversity and Biomonitoring, and were carried out in accordance with the guidelines and approval of the Animal Research and Ethics Committee of School of Life Science, South China Normal University (permissions, CAMC‐2018F). Genomic DNA was extracted from muscle tissue using a genomic DNA purification kit (Gentra Systems, Valencia, CA). The mtDNA cytochrome *b* gene and nuclear gene RAG‐1 region were amplified by polymerase chain reaction (PCR) using primers L14724 (5’‐GACTTGAAAAACCACCGTTG‐3’) and H15915 (5’‐CTCCGATCTCCGGATTACAAGAC‐3’) (Xiao et al., 2001), and RAG1F (5’‐CTGAGCTGCAGTCAGTACCATAAGATGT‐3’) and BR (5’‐CTGAGTCCTTGTGAGCTTCCATRAAYTT‐3’) (Lopez et al., 2004), respectively.

**TABLE 1:** Sampling localities, abbreviations, latitude and longitude, sample size, haplotype diversity, and nucleotide diversity for mitochondrial cyt *b* and nuclear RAG‐1 data of *Channa gachua*

Locality	*Abb*.	Latitude and longitude	Mitochondrial cyt *b*						Nuclear RAG−1					
			*n*	*N*a	Haplotype	*h*	*θπ*	*θω*	*n*	*N*a	Haplotype	*h*	*θπ*	*θω*
Bai Sha R.	SN1	18º41′*N*, 108º47′E	6	4	H1, H2, H3, H4	0.867	0.00175	0.00154	6	1	H1	–	–	–
Teng Qiao R.	SN2	18º35'*N*, 109º36'E	31	3	H37, H38, H39	0.185	0.00060	0.0011	31	5	H1, H61–H64	0.126	0.0001	0.0007
Ling Shui R.	SN3	18º41'*N*, 109º39'E	35	6	H23–H28	0.771	0.00292	0.00234	35	4	H1, H38–H40	0.084	0.00006	0.00041
Chang Hua R.	SN4	18º59'*N*, 109º36'E	29	10	H3, H5, H6, H7, H8, H9, H10, H11, H12, H13	0.850	0.01697	0.01072	29	6	H1–H6	0.591	0.0012	0.00188
Nan Du R.	NN1	19º09'*N*, 109º25'E	37	8	H29–H36	0.851	0.00436	0.00273	37	5	H1, H41–H44	0.306	0.0002	0.00068
Zhu Bi R.	NN2	19º18′*N*, 109º13′E	6	1	H48	1	0	0	6	2	H1, H121	0.485	0.0003	0.00022
Wan Quan R.	NN3	19º09'*N*, 110º18'E	40	10	H7, H20, H22, H37, H40–H45	0.827	0.01399	0.0099	40	7	H1, H3, H36, H37, H65–H67	0.602	0.001	0.00107
Long Shou R.	NN4	18º55′*N*, 110º19′E	10	5	H18, H19, H20, H21, H22	0.667	0.00164	0.00217	10	3	H1, H36, H37			
Yuan Jiang	MC1	23º16'*N*, 102º42'E	34	3	H14, H15, H16	0.558	0.00224	0.00193	33	41	H8–H10, H12–H14, H7–H9, H11–H13, H15, H17–H20, H29, H68–H96	0.983	0.0039	0.00416
Yi Luo Wa Di R.	MC2	25º13′*N*, 98º36′E	20	5	H14, H16, H17, H46, H47	0.742	0.00315	0.00297	20	34	H45, H53, H79, H86, H97–H120	0.973	0.0045	0.00295
Nu Jiang R.	MC3	24º09′*N*, 98º45′E	29	3	H15, H16, H17	0.640	0.00419	0.00223	25	16	H11, H45–H60	0.881	0.0043	0.00265
Lan Chang R.	MC4	23º02′*N*, 99º50′E	26	4	H14, H15, H16, H17	0.698	0.00354	0.0023	24	29	H7–H35	0.949	0.0039	0.00238
Ngang R.	V1	20º19'*N*,105º37'E	26	1	H51	0	0	0	7	1	H1	–	–	–
Khe Hói Dừa Stream	V2	16º08'*N*,107º41'E	7	2	H49, H50	0.476	0.00042	0.00036	26	1	H1	–	–	–
Lineage A1			170	37								–	–	–
Lineage A2			33	2		0.364	0.00524	0.00389				–	–	–
Lineage A3			24	6		0.951	0.01059	0.01141				–	–	–
Lineage B			109	6		0.780	0.00341	0.002				–	–	–
Overall			336	51		0.955	0.0318	0.01852	329	121		0.6913	0.0036	0.0064

Each 25 µl reaction mixture contained 2.5 ng of template DNA, 1 µl of each primer (10 nM), 9.5 μL ddH_2_O, and 12.5 μL of 2× Taq PCR Master Mix (Noweizan Co. Ltd, China). The PCR program run on a thermal cycler (Eppendorf Mastercycler) was as follows: one cycle of denaturation at 94°C for 2 min, 35 cycles of denaturation at 94°C for 45 s, annealing at 50–55°C for 45 s min, and extension at 72°C for 1 min 15 s followed by extension at 72°C for 10 min and holding at 4°C for storage. The purified PCR products were sequenced by Sangon Biotech Co., Ltd. (Shanghai) using an ABI PRISM 3730XL sequencer (Applied Biosystems, Foster City, CA, U.S.A.) with the BigDye Terminator Kit (Applied Biosystems). The chromatograms were assessed using CHROMAS software (Technelysium Pty Ltd, Australia), and the sequences were manually edited using BIOEDIT 6.0.7 (Hall, 1999).

### 2.2 Sequence alignment, phylogenetic analyses, and Bayesian species delimitation

The phase of the alleles was resolved computationally using PHASE v. 2.1.1. For the RAG‐1 gene, phylogenetic inference of independent alleles was also conducted on gamete phases by phase testing with DnaSP v.5.0 software (Librado & Rozas, 2009) with a probability threshold of 0.9 to resolve alleles. Sequences of the entire cyt *b* gene and RAG‐1 gene were aligned with Clustal X v2.1 (Thompson et al., 1997), and the evolutionary substitution models for mitochondrial (cyt *b*) and nuclear genes (RAG‐1) were the GTR + G + I model using the Akaike information criterion (AICc) in SMS (Smart Model Selection in PhyML) (Lefort et al., 2017).

A phylogenetic analysis was performed using the program BEAST 1.8.2 (Drummond et al., 2013) with 10^7^ MCMC steps, taking the first 10% as burn‐in and estimating the divergence times of the major lineages from the most recent common ancestor (TMRCA) by running 10^6^ generations. We employed TreeAnnotator v.2.2.1 (Rambaut & Drummond, 2015) in the BEAST package to summarize trees and displayed them using FigTree 1.4.3 (Rambaut, 2016) to display posterior probabilities (PP) and mean node ages for each node. A molecular clock in the cyt *b* gene was calibrated using a divergence rate of 1.05% per million years (Dowling et al., 2002). The minimum‐spanning tree was created via the MINSPNET algorithm, as implemented in Arlequin v3.5 (Excoffier & Lischer, 2010). We utilized the Bayesian phylogenetics and phylogeography (BPP) method to test the cryptic lineages of *C. gachua* (Yang, 2015). We performed the Bayesian phylogenetics and phylogeography (BPP) method under the multispecies coalescence model (MSC), as implemented in bpp 4.1.3 software (Yang, 2015). To examine whether the two lineages inferred from Cyt *b* trees represent different species, we used BPP with both cyt *b* and nuclear genes. We set the inverse‐gamma distribution G (3, 0.01) for theta and G (3, 0.004) for tau. We ran the MCMC analyses for 500,000 generations, sampled every five generations, and discarded samples from the first 50,000 generations as burn‐in. Each analysis was repeated at least twice to confirm consistency between runs. Topology based on Cyt *b* was used as a guide tree. We used the A10 model (species delimitation = 1, species tree = 0) and the species tree of the BEAST analyses (see below) as a user‐specified guide tree (Rannala & Yang, 2013; Yang & Rannala, 2010) treating the two distinct evolutionary lineages.

### 2.3 Population genetic structure

The genetic diversity of each population was estimated by using the number of haplotypes (*N*), haplotype diversity (*h*) (Nei & Tajima, 1983), and nucleotide diversity (*π*) (Jukes & Cantor, 1969) using DnaSP v5.0 software (Rozas et al., 2003). The existence of a phylogeographic structure was examined with two genetic differentiation indices (*G*_ST_ and *N*_ST_) following the method of Pons and Petit (1996) in software DnaSP v5.0 (Rozas et al., 2003). To quantify the genetic structure of *C. gachua* populations, we performed the pairwise *F*_ST_ values and AMOVA (analysis of molecular variance) by Arlequin version 3.5 (Excoffier & Lischer, 2010). AMOVA was used to partition variation among samples into within‐population (*F*_ST_), within‐group (*F*_SC_), and among‐group (*F*_CT_) components to evaluate the most likely population configuration and geographical subdivision with K_2_P distance and 20,000 permutations. The table was prepared using R v.3.2.4. For the hierarchical analysis, populations according to the different geographical barriers were grouped together to investigate the potential effects of various geographical barriers: (1) Two geographical groups were primarily divided by the Gulf of Tonkin: Hainan Island + Vietnam and mainland China; (2) two geographical groups were primarily divided by the Gulf of Tonkin: Hainan Island and Vietnam + mainland China; (3) three geographical groups were divided according to the three geographical regions: Vietnam, Hainan Island, and mainland China; and (4) four geographical groups were primarily divided by the WY Range in the Hainan region, Vietnam, and mainland China. The program SAMOVA (Dupanloup et al., 2002) was also employed to delineate the best clustering of population groups with the maximum extent of genetic differentiation. These analyses employed 500 simulated annealing steps and compared maximum indicators of differentiation (*F*_CT_) when the program was instructed to identify *K* = 2–6 potential population units.

### 2.4 Historical demography, divergence time estimation, and biogeographic analysis

We implemented neutrality tests (Tajima's *D* test (Tajima, 1989) and Fu's *F*s test (Fu, 1997)) and mismatch distributions to assess signatures of recent historical demographic events using DnaSP v5.0 software (Librado & Rozas, 2009). Bayesian skyline plot (BSP) analyses for the effective population size changes over time and the time to the most recent common ancestor (TMRCA) for each lineage were evaluated in BEAST v1.8.2 for *C. gachua* (Drummond et al., 2013). Two independent MCMC simulations ran for 200,000,000 generations to ensure convergence of all parameters (ESSs > 200); the first 10% of samples for each chain were discarded as burn‐in. Bayesian skyline plots (BSP) and TMRCA analysis were plotted using Tracer v1.6 (Rambaut et al., 2014). A 1.05%/MY divergence rate has been calibrated for the mtDNA cyt *b* genes in multiple bony fishes for population expansion (Dowling et al., 2002).

To further discover the historical biogeography of *C. gachua*, ancestral areas were reconstructed using statistical dispersal–vicariance (S‐DIVA) in RASP 3.2 (Yu et al., 2015). The evolutionary histories were composed of events such as vicariance, dispersal, extinction, or standard speciation. Four areas were defined for the biogeographic analyses according to the sampling and distribution range of *C. gachua*: (1) the mainland China region (M: MC1, MC2, MC3, and MC4); (2) the southern Hainan region (S: SN1, SN2, SN3, and SN4); (3) the northern Hainan region (N: NN1, NN2, NN3, and NN4); and (4) the Vietnam region (V: V1 and V2).

## 3 RESULTS

### 3.1 Genetic diversity of *Channa gachua*

A total of 1,140 base pairs (bp) of mtDNA cyt *b* gene sequences from 336 specimens and 1,194 base pairs (bp) of nuDNA RAG‐1 gene sequences from 329 specimens were analyzed. All sequences of the nuDNA RAG‐1 gene were phased, and the final datasets with inferred phased sequences consisted of 658 sequences for *C. gachua*. The obtained sequences were deposited in GenBank under accession numbers for cyt *b*: MW233899‐MW233949 and for RAG‐1: MW233950‐MW234070. The nucleotide sequences in the mtDNA cyt *b* gene were A + T‐rich (55.5%), which consisted of 13.8% guanine, 25.3% adenine, 30.2% thymine, and 30.7% cytosine, and the nuDNA RAG‐1 gene was not A + T‐rich (45.8%), which consisted of 27.8% guanine, 26.4% adenine, 21.9% thymine, and 23.9% cytosine. A total of 51 haplotypes (127 variable sites and 120 phylogenetically informative sites) in the mtDNA cyt *b* gene and a total of 122 haplotypes (66 variable sites and 32 phylogenetically informative sites) in the nuDNA RAG‐1 gene were obtained. Among the 51 haplotypes, only five (haplotypes H03, H07, H20, H22, and H37) were shared by populations SN2, SN3, SN4, and NN3 on Hainan Island, and four (haplotype H14‐H17) were shared by populations MC1, MC2, MC3, and MC4 in mainland China in the mtDNA cyt *b* gene (Table 1). Overall, the total haplotype diversity was high (0.955), ranging from 0.000 (V1) to 0.867 (SN1), and the nucleotide diversity within *C. gachua* was low (0.0318), ranging from 0.000 (NN2 and V1) to 0.0169 (SN4) (Table 1). The Hainan Island region exhibited the highest total haplotype diversity (0.951) among all regions followed by the mainland China region (0.780) and the Vietnam region (0.364) (Table 1).

### 3.2 Phylogenetic reconstruction and Bayesian species delimitation

**FIGURE 2:**
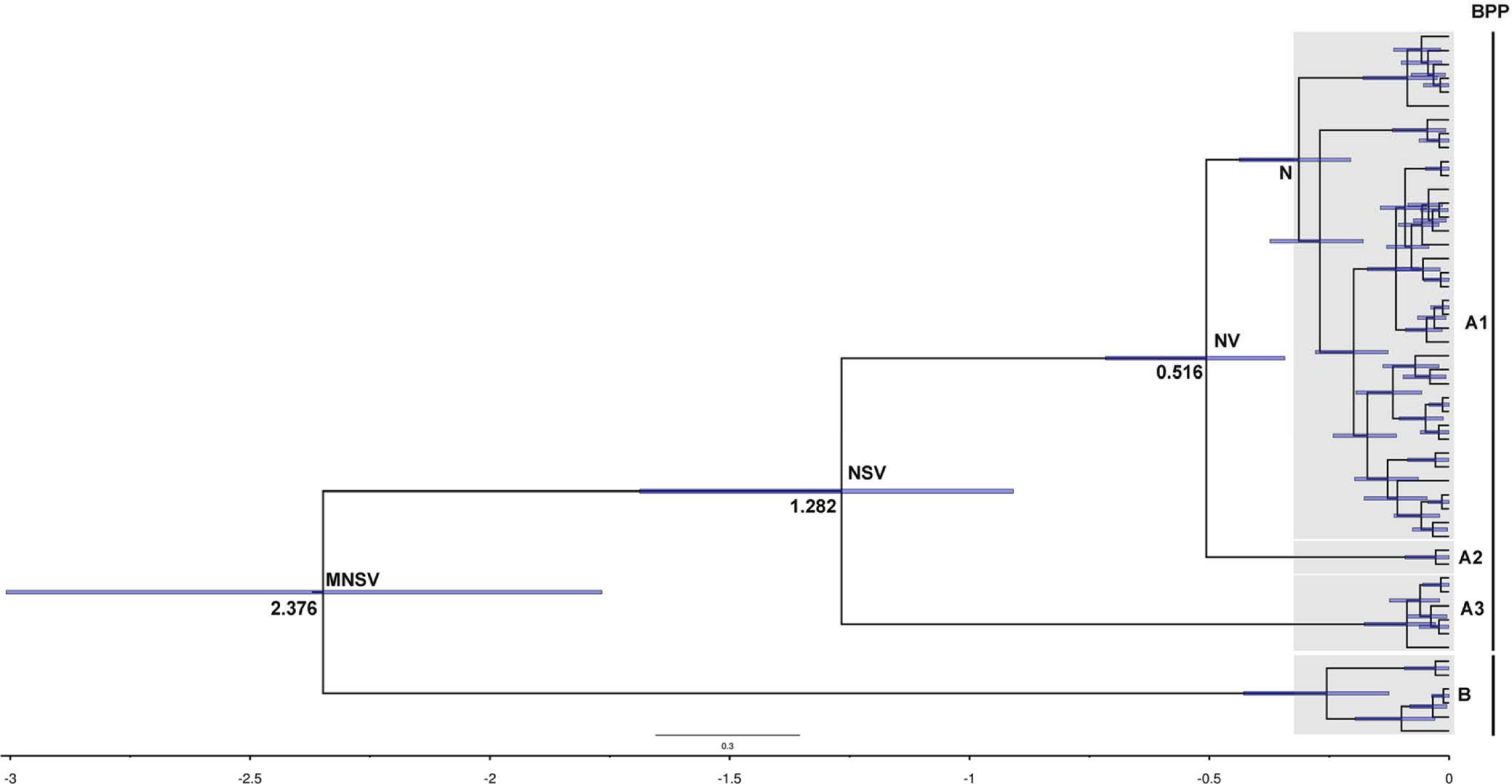
Phylogenetic tree of genetic relationships based on mitochondrial cyt *b* among 14 populations in *Channa gachua* using 51 haplotypes. The results are also presented based on Bayesian phylogenetics and phylogeography (BPP).✩: indicated vicariance events; ↘: indicated dispersal events; M: mainland China region; S: southern Hainan region; N: northern Hainan region; V: Vietnam region

**FIGURE 3:**
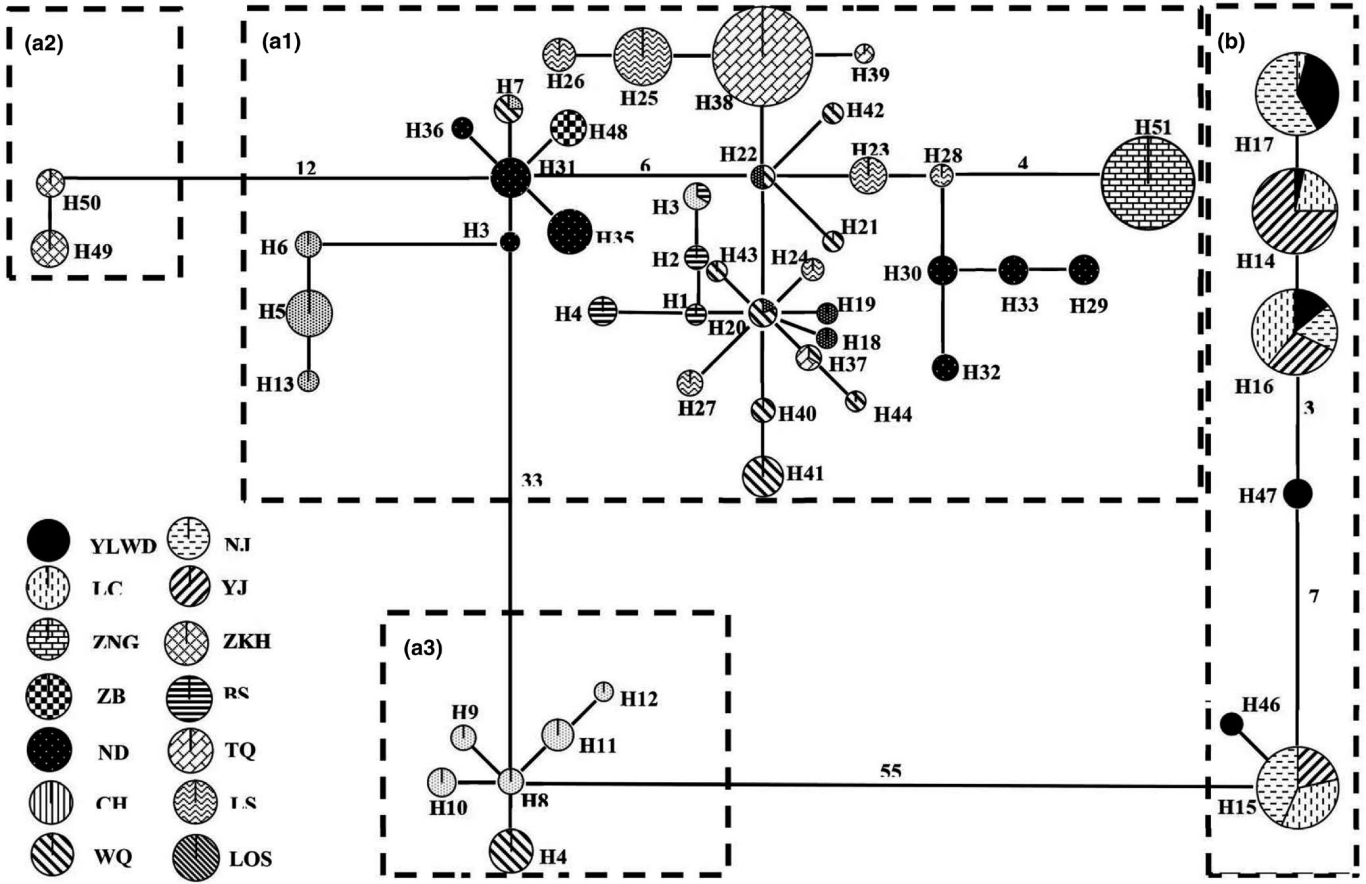
Minimum spanning network (MSN) based on mutations between haplotypes observed in populations of *Channa gachua*. Haplotype designations (Table 1) are indicated next to each circle. Locality designations (see Figure 1) for specimens possessing each haplotype are indicated inside the circles. The sizes of the circles are proportional to the number of individuals represented. The length of the lines between circles is roughly proportional to the estimated number of mutational steps between the haplotypes

A comparison between the *N*_ST_ and *G*_ST_ fixation indices demonstrated that *N*_ST_ was larger than *G*_ST_ (0.879 and 0.352, respectively), demonstrating the presence of phylogeographic structure in *C. gachua* (Pons & Petit, 1996). The topological relationships obtained from the phylogenetic analysis support the formation of four major lineages (A1, A2, A3, and B) that were recovered according to the distribution pattern from different populations based on the mtDNA cyt *b* gene (Figure 2). For the phylogenetic analyses, the topology of BEAST and haplotype networks were recovered from the same tree topologies. Lineage A1 was distributed in most populations from Hainan Island and one population (V1) from Vietnam. Lineage A2 was only composed of one population (V2) and was also found in Vietnam. Lineage A3 was composed of the individuals from SN4 and NN3 on Hainan Island, and lineage B included specimens from populations MC1, MC2, MC3, and MC4 in mainland China (Figure 2). The network also supported the notion that all mtDNA haplotypes fell into four major lineages (A1, A2, A3, and B), with lineages A1 and A3 being located in the interior and the others being located at the tip (Figure 3). The phylogenetic tree showed that all sequences fell within three allopatric lineages (Ⅰ, Ⅱ, and Ⅲ) based on the nuDNA RAG‐1 gene. Lineage Ⅰ was composed of individuals from Hainan Island and Vietnam, and lineages Ⅱ and Ⅲ included specimens from mainland China (Figure S1).

Species delimitation analysis in BPP delimited 2 divergent mitochondrial and nuclear lineages of *C. gachua* as separate species. Bayesian phylogenetics and phylogeography (BPP) and minimum‐spanning networks were largely concordant. Lineage A was distributed on Hainan Island and Vietnam as a separate species, and the specimens from mainland China (lineage B) were delimited as a separate putative species (Figure 2).

### 3.3 Population genetic structure of *C. gachua*

The value of overall *F*_ST_ was 0.711, and the range of pairwise *F*_ST_ values between populations varied from 0.020 (between MC3 and MC4 populations) to 1.000 (between NN2 and V1 populations) in mtDNA cyt *b* gene. Moreover, the pairwise *F*_ST_ values between populations from different regions were relatively high and significant, indicating that high differentiation existed among these populations (Table 2). Hierarchical analyses of molecular variance (AMOVA) from the two geographical regions (Hainan Island + Vietnam and mainland China) demonstrated that significant spatial genetic structuring among groups was 85.81% (*F*_CT_ = 0.858, *p* < .000) but was only 5.85% (*F*_SC_ = 0.412, *p* < .000) among populations within groups and 8.34% (*F*_ST_ = 0. 917, *p* < .000) within populations (Table 3). However, AMOVA from the two geographical regions (Hainan Island and Vietnam + mainland China) identified only 60.49% of the variants to be present among groups, 28.70% of the variation among populations within groups, and 10.81% of the variation within populations (Table 3). This result indicated that the Gulf of Tonkin was a geographical barrier between Hainan Island and mainland China but not between Vietnam and Hainan Island (Table 3). When the 14 populations were divided into *K* = 3 groups, (1) Hainan Island and Vietnam, (2) the V2 population in Vietnam, and (3) mainland China revealed the highest *F*_CT_ value (0.858, *p* < .01) based on estimates of the SAMOVA.

**TABLE 2:**
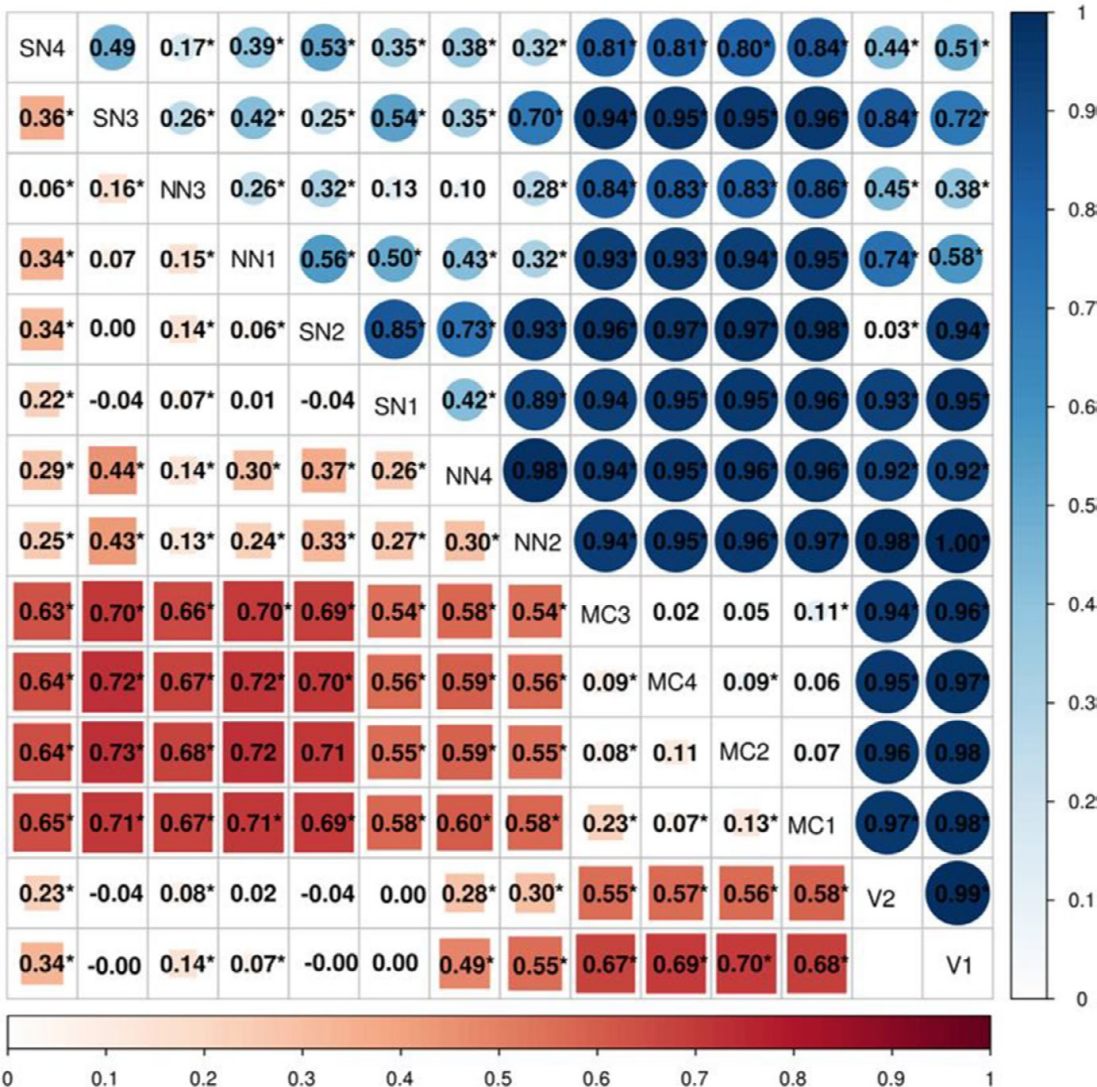
Matrix of pairwise *F*_ST_ among fourteen populations based on nuclear RAG‐1 (below diagonal) and mitochondrial cyt *b* (above diagonal) in *Channa gachua*

Note

*p < .05 for indices of population differentiation, *F*_ST_.

**TABLE 3:** Analysis of molecular variance (AMOVA) for *Channa gachua* based on mtDNA

Scheme Category description	% Var.	Statistic	*p*
**Two geographical groups (Hainan island + Vietnam) (mainland China)**			
Among groups	**85.81**	*F*_SC_ = 0.412	.000
Among populations within groups	5.85	*F*_ST_ = 0.917	.000
Within populations	8.34	*F*_CT_ = 0.858	.000
**Two geographical groups (Hainan island)(Vietnam + mainland China)**			
Among groups	60.49	*F*_SC_ = 0.726	.000
Among populations within groups	28.70	*F*_ST_ = 0.892	.000
Within populations	10.81	*F*_CT_ = 0.605	.007
**Three geographical groups(Hainan island) (Vietnam) (mainland China)**			
Among groups	**83.27**	*F*_SC_ = 0.397	.000
Among populations within groups	6.64	*F*_ST_ = 0.899	.000
Within populations	10.09	*F*_CT_ = 0.833	.000
**Four geographical groups (Vietnam) (mainland China) (NN1 + NN2 + NN3 + NN4) (SN1 + SN2 + SN3 + SN4)**			
Among groups	**78.95**	*F*_SC_ = 0.405	.000
Among populations within groups	8.53	*F*_ST_ = 0.875	.000
Within populations	12.51	*F*_CT_ = 0.789	.000
**SAMOVA *K*** = **3 (Hainan island + V1) (mainland China) (V2)**			
Among groups	86.87	*F*_SC_ = 0,383	.000
Among populations within groups	5.41	*F*_ST_ = 0.913	.000
Within populations	8.72	*F*_CT_ = 0,858	.000

### 3.4 Historical demographic expansion, molecular dating, and historical biogeography

According to the neutral test, the values of Tajima's *D* and Fu's *F*s tests were positive, and multimodal mismatch distributions were observed for all three clades (Table 1). These results all did not support population expansion for *C. gachua*. The Bayesian skyline plot (BSP) analyses also indicated a decline in effective population size 75,000 years ago (75 Kya) (Figure S2).

The time to coalescence, estimated using the BEAST analyses, was 2.376 myr in *C. gachua*. Molecular dating estimated that sublineage A1 + A2, lineage A and lineage B coalesced to TMRCA 0.516 (95% highest posterior density (HPD): 0.343–0.716) and 1.282 (95% HPD: 0.909–1.688) myr ago, respectively. The results of the S‐DIVA analysis indicated that the common ancestor of *C. gachua* was distributed on Hainan Island, Vietnam, and mainland China with an occurrence frequency of 100%. Hainan Island was isolated from mainland China and separated the populations into two groups, that is, south and north of the Gulf of Tonkin. Two vicariance events occurred: The rise of the WY Range on Hainan Island separated the populations into two groups (lineage A1+A2 and lineage A3), and the formation of the Qiongzhou Strait separated the Hainan Island population (lineage A1) and the Vietnam population (lineage A2). After a vicariance event in the formation of the WY Range, the populations in the South Hainan region moved northward to the North Hainan region (Figure 2).

## 4 DISCUSSION

### 4.1 High population differentiation and low diversity of *Channa gachua*

For conservation planning for freshwater fishes, the application of genetic diversity to reflect the ability of populations to adapt to environmental factors has been presented over the last several decades (Bernatchez, 2016). Our study, using both mitochondrial and nuclear markers, demonstrated that genetic variation is relatively high in *C. gachua*. The total haplotype diversity (*h*) across the samples was 0.955, indicating a high level of genetic diversity in *C. gachua*, which is similar to the results obtained for other freshwater fishes in South China, such as *G. hainanensis* (*h* = 1.00; Chen et al., 2007), *G. orientalis* (*h* = 0.981; Yang et al., 2016), and *O. hainanensis* (*h* = 0.946; Zhang et al., 2020). Compared with other freshwater fishes in South China, the nucleotide diversity of *C. gachua* (*θπ* = 3.18%) was higher than that of other species (1.494% for *O. hainanensis*, see Zhang et al., 2020; 1.49% for *A. normalis*, see Huang et al., 2019; 2.292% for *O. lepturum*, see Zhou et al., 2017; 0.744% for *G. orientalis*, see Yang et al., 2016; and 1.875% for *G. hainanensis*, see Chen et al., 2007). However, the nucleotide diversity value (*θπ* = 1.05%) of the *C. gachua* populations on Hainan Island was similar and comparable to the results reported for *O. hainanensis* (*θπ* = 1.63%) (Zhang et al., 2020) and *G. orientalis* (*θπ* = 0.77%) (Yang et al., 2016), but higher than that of the mainland China and Vietnam populations (0.341% and 0.524%, respectively). In general, refugial populations could harbor considerably higher levels of genetic diversity and private haplotypes than migratory populations (Avise, 2000). Therefore, the population on Hainan Island, which exhibited the highest genetic diversity, may have served as a genetic reservoir for *C. gachua*, from which complex colonization scenarios could have originated. Our study also suggested that *C. gachua* possesses a low level of genetic diversity in a single population (*θπ* = 0.04%–1.39%), which is suspected to cause genetic drift by effective population size decline and a high level of genetic diversity in the total population (*θπ* = 3.18%). As in previous studies, the reason for effective population size decline in freshwater fish has occurred not only due to overfishing but also due to massive loss of their natural habitat in mainland China (Kang et al., 2014). Similarly, overfishing and anthropogenic disturbance were also suggested to be major factors leading to population declines in *C. gachua* on Hainan Island and mainland China (Kang et al., 2014).

Moreover, the population analyses of *C. gachua* displayed a significant relationship between phylogeny and geography (*N*_ST_ = 0.879 and *G*_ST_ = 0.352), demonstrating the presence of phylogeographic structure. *Channa gachua* displayed a high level of genetic differentiation (*F*_ST_ = 0.876), with the most unique haplotypes and partially shared haplotypes demonstrating that the populations of *C. gachua* were differentiated from each other (Figure 3). Such a high level of genetic differentiation resulted from the different haplotype compositions among populations and regions. According to the landscape of Hainan Island, rivers mostly originate from the central mountainous area with high elevation, characterized by a radial river system. The topography is characterized by mountains in the central part of Hainan Island, and neighboring rivers are not connected to each other. Our study showed that genetic differentiation is relatively high in comparison with those of other sympatric freshwater fishes, regardless of their status as downstream species; these fishes include *A. normalis* (*F*_ST_ = 0.530; Huang et al., 2019), *O. lepturum*, (*F*_ST_ = 0.750; Zhou et al., 2017), and *Carassioides cantonensis* (*F*_ST_ = 0.036; Deng et al., 2014). This result indicates that the effects of geographical barriers are more significant on upstream fishes than downstream fishes. In other words, species exhibit more restricted gene flow in the headwaters of a drainage system compared with more downstream species. Thus, high genetic differentiation between population in different rivers is expected. *F*_ST_ values were highest between the mainland China populations (MC1, MC2, MC3, and MC4) and all other populations (*F*_ST_ between 0.805 and 0.976), suggesting strong genetic differentiation and consisting of two cryptic species (described below).

According to the SAMOVA results and the partitioning of genetic variation through AMOVA, 85.81% of the total genetic variation in *C. gachua* could be attributed to genetic differences between the two geographical regions (Hainan Island + Vietnam and mainland China), whereas only 5.85% variation was observed among populations within groups. However, 83.27% of the total genetic variation among the three geographical regions (Hainan Island, Vietnam, and mainland China) may indicate that the Gulf of Tonkin is not a major geographical barrier between Hainan Island and Vietnam for *C. gachua*. The genetic data suggest that this current spatial distribution was achieved relatively recently, given that populations had weak genetic differentiation between Hainan Island and Vietnam, supporting the occurrence of enabling mixing of populations across this ocean barrier (Gulf of Tonkin). To the best of our knowledge, this report describes the first time that the Gulf of Tonkin has not represented an important geographical barrier between Hainan Island and Vietnam for freshwater fish.

### 4.2 History demography

Glacial cycles during the Pleistocene significantly influenced the demographic history of freshwater fishes in mainland China, such as *Rhodeus ocellatus* (Yang et al., 2018), *Squalidus argentatus* (Yang et al., 2012), *G. orientalis* (Yang et al., 2016), and *O. hainanensis* (Zhang et al., 2020). However, neutral tests, mismatch distribution analyses, and Skyline plots indicated that range expansion did not occur in *C. gachua*. Moreover, a Bayesian skyline plot (BSP) indicated a decline in effective population size 75,000 years ago (75 Kya), coinciding with the last glacial period in relation to sea‐level rise in the Gulf of Tonkin. We suggest that Hainan Island is located in the transitional zone between the subtropical and tropical zones and that *C. gachua* exhibits a more restricted habit in the headwaters of a drainage system. During glacial cycles leading to sea‐level fluctuations, the habitat area will not increase for *C. gachua*, which indicates that the effects of population expansion are more significant for downstream fishes than for upstream fishes. Therefore, the results of demographic analysis of *O. hainanensis* in downstream fish displayed a pattern of population expansion (Zhang et al., 2020), but that of *C. gachua* did not.

### 4.3 Phylogeography of *Channa gachua*

According to all results, our study suggests that the ancestral populations of *C. gachua* were distributed in mainland China, Vietnam, and Hainan Island before the Pleistocene (2.376 myr). During glaciations after the warmer Miocene and Pliocene, marine transgressions were extensive, and most of the continental shelves of the South China Sea, including the Gulf of Tonkin, were underwater (Duan & Yongyang, 1989; Liu, 1994; Voris, 2000; Zhao et al., 2007). However, the ancestral populations of *C. gachua* were distributed in mainland China and Hainan Island before the Pleistocene, and mainland China and Hainan Island were connected as the Hainan Peninsula (Zeng & Zeng, 1989). Thus, the gene flow of *C. gachua* between mainland China and the southern Hainan Peninsula (South China Sea) was absent during glaciations.

Approximately between 2 and 2.5 myr, Hainan was first isolated from the mainland due to volcanism and sinking land in the current Gulf of Tonkin (e.g., Zeng & Zeng, 1989; Zhao et al., 2007). After the formation of the Gulf of Tonkin, the gene flow of *C. gachua* between mainland China, Vietnam, and southern Hainan Island was restricted during Pleistocene glaciations. The ancestral populations of *C. gachua* dispersed from southern Hainan Island to northern Hainan Island, including the Changhua River and Wanquan River (lineage A3) (Figure 2). After the formation of the WY Range, the populations of *C. gachua* were divided into two groups, South Hainan Island and North Hainan Island. Based on this grouping scheme, our study proposed two geographical barriers: the central mountainous area of Hainan Island (WY Range) and the Gulf of Tonkin (Figure 2).

The WY Ranges are located in the central and southern regions of Hainan Island and approach an elevation of 1,800 m. Geological studies (Duan & Yongyang, 1989; Xie et al., 2012) indicate that the uplift of the WY Ranges promoted the development of habitable river systems on Hainan Island and that seawater inundation caused isolation of the North Hainan populations. Despite repeated land connections between the mainland and Hainan at various times since the Miocene, the North Hainan Island populations do not seem to have ever rejoined the western mainland or the South Hainan Island populations in a manner that enabled gene exchange. Accordingly, the populations on South Hainan Island were first isolated as lineage A3. These results also supported the phylogenetic tree (Figure 2). These results also showed that the WY Ranges were raised before the Qiongzhou Strait formed.

Previous studies (e.g., Zhang et al., 2020) all supported that the Gulf of Tonkin was an important barrier limiting gene exchange between populations on Hainan Island and mainland China in interglacial periods, but our study found that the Gulf of Tonkin, not serving as an important barrier, also isolated the gene flow of *C. gachua* between mainland China and Hainan Island during glaciations. This result is surprising because the population in Vietnam is separated from other populations by a relatively long distance. The lack of clear genetic differentiation of *C. gachua* between Vietnam (V1) and Hainan Island in both types of DNA markers could be caused by recent large‐scale dispersal and colonization events. We suggest that the fall in sea levels would result in the Gulf of Tonkin becoming terrestrial along with the old Red River drainage system, which appears to have existed during periods of glacial maxima. According to the phylogenetic tree, the V2 population in Vietnam formed a monophyletic group and was located in the basal position of lineage A. We suggest that rising sea level, during glacial retraction, would flow on the Gulf of Tonkin from the south direction and the V2 population located in the southern part of the Gulf of Tonkin was the first population to be isolated geographically after the retreat of glaciation. The monophyly of *O. hainanensis* populations in the Changhua River and Red River indicated a close relationship of populations between Hainan Island and mainland China (Zhang et al., 2020). According to habitat and ecology, *C. gachua* occurs on the bottom of fast‐flowing mountain streams. Thus, although the Gulf of Tonkin was exposed to glaciations, the populations of *C. gachua* were not distributed. Only the floods may have created temporary confluences allowing dispersal. Thus, our study suggests that the phylogeographic pattern of *C. gachua* was different from that of other fishes due to its habits.

### 4.4 Molecular species delimitation

Several molecular studies suggest two cryptic species in *C. gachua*. Phylogenetic analyses of the mtDNA Cyt *b* gene demonstrated that *C. gachua* is composed of two distinct lineages that exhibit deep genetic divergence, which is a well‐established criterion for species delimitation. The results obtained for the nuclear RAG‐1 gene that we examined were in keeping with the mtDNA finding, indicating that lineages A1‐A3 and B are two cryptic lineages/species. Based on these nuclear and mitochondrial data, our study strongly suggests that there are two cryptic species distributed on Hainan Island (belonging to the Hainan Island zoogeographical region) and mainland China (belonging to the Nujiang–Lancangjiang zoogeographical region). Four well‐supported evolutionary lineages were identified in the mtDNA trees, which may include two cryptic species (lineages A1‐A3 and lineage B) (Figure 2). Moreover, the nuDNA trees also presented two divergent lineages, which was consistent with the mtDNA trees (Figure S1). The haplotype networks of *C. gachua* demonstrated the same pattern as the phylogenetic analysis with 55 mutation steps between the two major lineages (lineages A and B) (Figure 3), implying deep divergence between them. The species delimitation method (BPP) was used to infer cryptic species in *C. gachua* (Figure 2). The genetic differentiation (*F*_ST_) between the two cryptic species was 0.916, which is also indicative of high differentiation between them (Balloux & Lugon‐Moulin, 2002).

### 4.5 Conservation implications

According to the model proposed by Moritz, evolutionarily significant units (ESUs) are recommended on the basis of reciprocal mtDNA monophyly, while management units (hereafter MUs) have been defined as geographical areas with restricted interchange in allele frequency distributions in mitochondrial or nuclear loci (Moritz, 1994). Understanding genetic variability and phylogeography can contribute to the development and management of effective conservation programs. The importance of fish diversity protection is generally considered in South China, but there are few studies on the basis of fish diversity. In this study, two unique evolutionary lineages (A and B) within *C. gachua* were represented and should be considered two cryptic species (Figure 2). The importance of recognizing cryptic *C. gachua* species has implications for the assessment of conservation. The two cryptic species may represent a means of improving future conservation plans, which should be aimed at managing them independently; furthermore, they should be considered two ESUs. We suggest that protection is required for synchronization with the genetic uniqueness of the different MUs. In our study, some populations in the Changhua and Wanquan Rivers (lineage A3) were the first to differentiate as independent sub‐branches, and their *F*_ST_ was significantly high, suggesting that they should be regarded as different MUs. We also suggest that two lineages (A1–A2) should be regarded as different MUs distributed in the other population of Hainan Island and the population in Vietnam.

## CONFLICT OF INTEREST

The authors declare no competing interests in the preparation or execution of this study.

## AUTHOR CONTRIBUTIONS

**Junjie Wang:** Data curation (equal); Investigation (equal); Writing‐original draft (equal). **Chao Li:** Data curation (equal); Investigation (equal); Methodology (equal). **Jiaqi Chen:** Methodology (equal). **Jujing Wang:** Investigation (equal). **Jinjin Jin:** Investigation (equal). **Shuying Jiang:** Investigation (equal). **Luobin Yan:** Investigation (equal). **Hung‐Du Lin:** Conceptualization (equal); Writing‐original draft (equal); Writing‐review & editing (equal). **Jun Zhao:** Conceptualization (equal); Funding acquisition (lead); Project administration (lead); Resources (equal); Writing‐review & editing (equal).

## Supporting information

Fig S1

Fig S2

## Data Availability

The data that support the findings of this study are available in GenBank (https://www.ncbi.nlm.nih.gov/), reference number (cyt *b*: MW233899‐MW233949 and RAG‐1: MW233950‐MW234070). Details regarding individual samples are available in Table 1.
