# Supplementary figures and images for: Phylogeographic structure of the dwarf snakehead (Channa gachua) around Gulf of Tonkin: Historical biogeography and pronounced effects of sea‐level changes
Source: Ecol Evol. 2021 Aug 17;11(18):12583–95. doi: 10.1002/ece3.8003 (PMC8462176; doi:10.1002/ece3.8003)

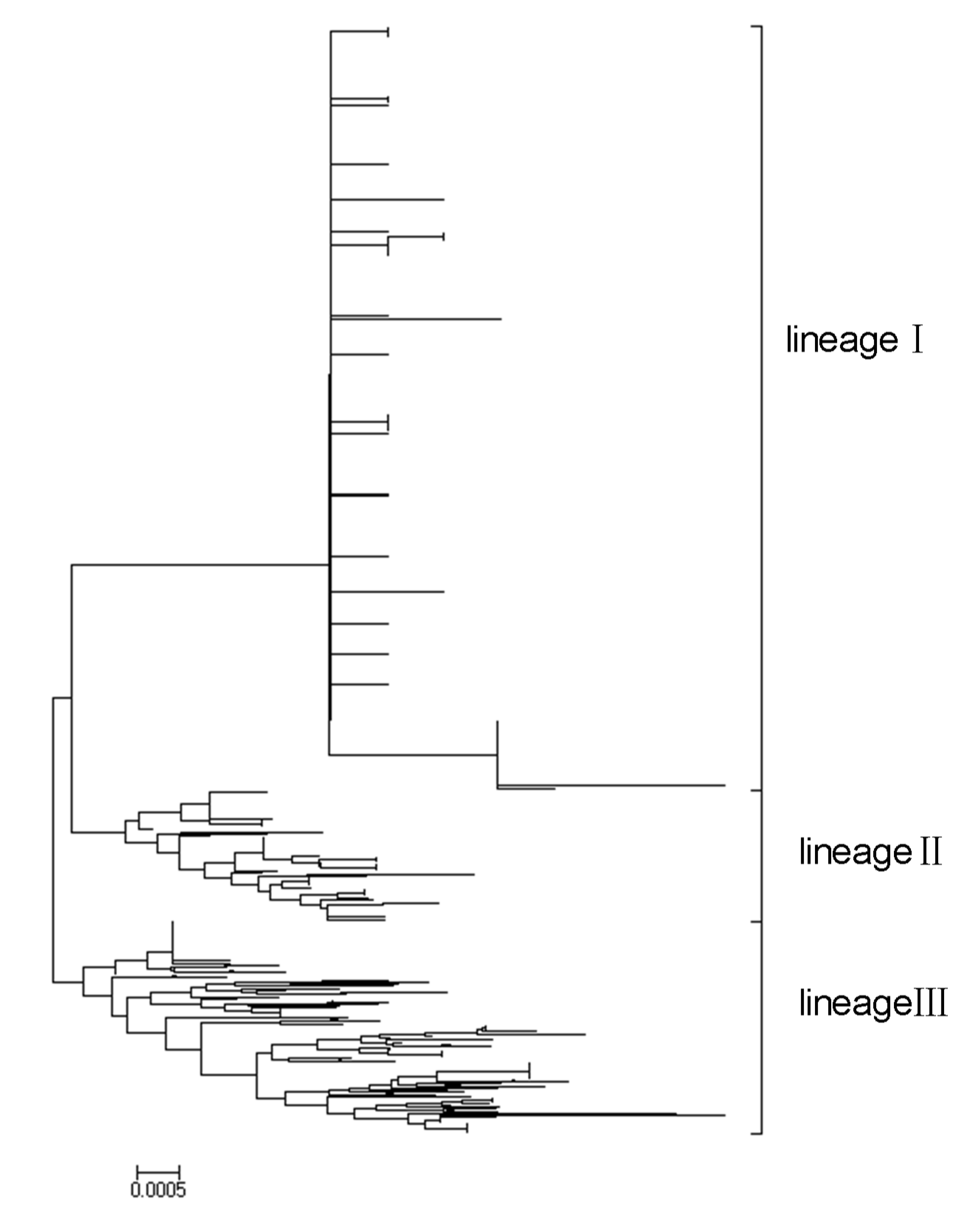

Supplement: Supplementary file 1 — Fig S1 [file ECE3-11-12583-s002.tif]

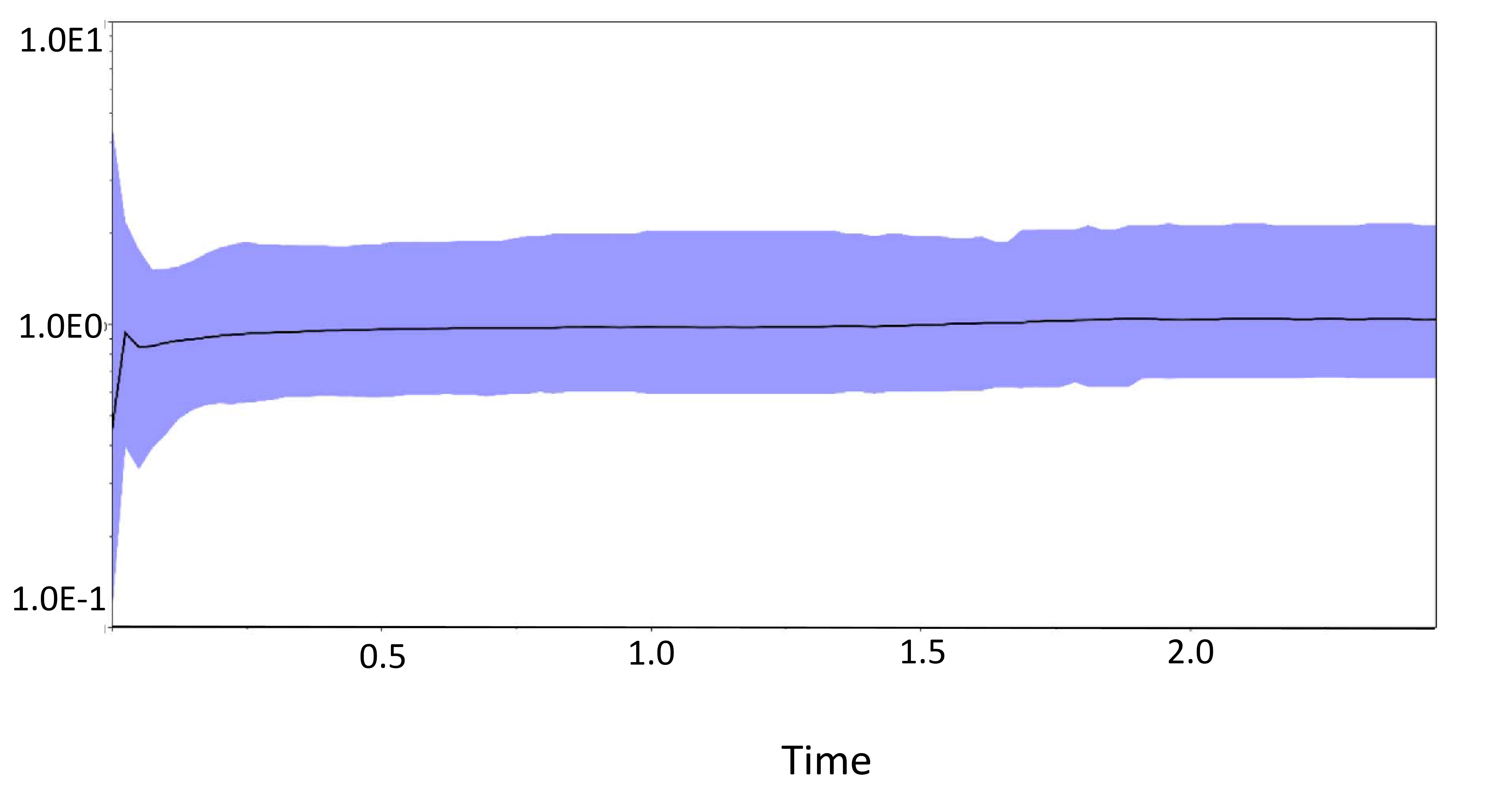

Supplement: Supplementary file 2 — Fig S2 [file ECE3-11-12583-s001.tif]
